# Adult versus Pediatric Neuroblastoma: The M.D. Anderson Cancer Center Experience

**DOI:** 10.1155/2014/375151

**Published:** 2014-06-12

**Authors:** Henry J. Conter, Vancheswaran Gopalakrishnan, Vinod Ravi, Joann L. Ater, Shreyaskumar Patel, Dejka M. Araujo

**Affiliations:** ^1^Division of Cancer Medicine, M.D. Anderson Cancer Center, 1400 Holcombe Boulevard, Unit 463, Houston, TX 77030, USA; ^2^Department of Sarcoma Medical Oncology, M.D. Anderson Cancer Center, 1400 Holcombe Boulevard, Unit 463, Houston, TX 77030, USA; ^3^Department of Pediatrics Patient Care, M.D. Anderson Cancer Center, 1400 Holcombe Boulevard, Unit 463, Houston, TX 77030, USA

## Abstract

*Background*. Staging and treatment of adult neuroblastoma has yet to be formalized. We sought to determine the utility of the pediatric classification system in adults and determine the efficacy of different treatment modalities.* Methods*. Medical records of 118 adults (patients >17 years old) and 112 pediatric patients (ages 2–17), who were treated for neuroblastoma at M.D. Anderson Cancer Center from January 1994 to September 2012, were reviewed. International neuroblastoma risk group (INRG) variables were abstracted. The primary outcome of interest was actuarial progression-free survival.* Results*. Median age of pediatric patients was 5 years (range 3–16) and 47 years (range 18–82) for adult patients. There were no differences in PFS or OS between stage-matched risk categories between pediatric and adult patients (L1-*P* = 0.40, L2-*P* = 0.54, and M-*P* = 0.73). In the treatment of L1 disease, median PFS for adults treated with surgery and radiation was 11.1 months compared with single modality local treatment ± chemotherapy (6.4 and 5.1 months, resp.; *P* = 0.07). Median PFS in L2 adult patients was 5.2 months with local therapy and 4 months with the addition of chemotherapy (*P* = 0.23).* Conclusions*. Adult and pediatric patients with neuroblastoma achieve similar survival outcomes. INRG classification should be employed to stratify adult neuroblastoma patients and help select treatment.

## 1. Introduction 

Neuroblastoma arises from primitive sympathetic neural cells primarily in the adrenal medulla and also in the paraspinal sympathetic ganglia in the neck, chest, abdomen, or pelvis [1]. Although neuroblastoma currently represents 7% of all childhood malignancies or roughly 1 case per 100,000 children per year, only 1 case per 10 million adults per year is diagnosed in adulthood [2–4]. Because of the rarity of adult neuroblastoma, staging systems and risk assessment tools have been developed using primarily pediatric data [5]. Clinically relevant pediatric factors that influence survival in children include stage, age, histology, tumor grade, MYCN oncogene status, chromosome 11q status, and DNA ploidy. These factors are currently part of the international neuroblastoma risk assessment system [6].

Whereas 5-year survival approaches 85% for infants diagnosed with neuroblastoma, adult overall survival at 5 years is 36% [4]. Long-term survival in adults has been reported even in the presence of multiple recurrences [7]. Up to 1/3 of adult patients are diagnosed with metastatic disease at presentation. In the metastatic setting, prognosis between adults and children appears comparable. However, at earlier stages, the prognosis of adults is worse than that of comparable pediatric patients. Adult patients have been reported to have a high incidence of unfavorable histologies [8] but a lower incidence of MYCN amplification [9, 10], which usually correlates with a better prognosis.

No standard therapy exists for adults with neuroblastoma, but all adult patients can be considered at high risk of death from disease regardless of stage [11]. Dose intense cytotoxic chemotherapy has been shown to produce greater response rates and progression-free survival, compared to standard chemotherapy [12, 13]. Commonly used agents include cisplatin, cyclophosphamide, ifosfamide, topotecan, etoposide, doxorubicin, and vincristine. High-dose chemotherapy and autologous stem-cell rescue (HDCSR) have been shown to improve event-free and overall survival in high-risk pediatric patients, based on a meta-analysis of 3 randomized controlled trials including 739 patients [14]. External beam radiation may also play a role in the management of high-risk neuroblastoma, especially in the setting of residual disease after surgery [15]. However, adult patients may have poorer tolerance to high intensity therapy regimens compared to children. For patients with metastatic neuroblastoma, radiation therapy can be an effective treatment modality when local control is required but may hinder attempts at gross total resection of the tumor [16].

Although there has been a trend to improved prognosis of patients over the last three decades [4], only 3 adult case series since 1997, totaling 93 patients, have been published [8–10]. We sought to improve on the volume of the literature on this subject by reviewing the M.D. Anderson Cancer Center experience of treating adult patients with neuroblastoma and comparing outcomes to their pediatric counterparts.

## 2. Methods

### 2.1. Study Protocol

Patients were identified by query of the University of Texas M.D. Anderson Cancer Center's (MDACC) institutional database from its inception in January 1994 until the date of search, September 21, 2012. All patients diagnosed by histology with neuroblastoma or ganglioneuroblastoma were eligible for inclusion into the study. However, patients who were 2 years of age or younger at diagnosis were excluded from analysis to limit the effect of spontaneous regression [17]. Adults were defined by convention by being diagnosed after the age of 17. The institutional database routinely captures demographic, histologic, location of the primary tumor, SEER stage, and vital statistic information on all patients seen at MDACC. Data were collected from the database using a standardized form. Additional information concerning the international neuroblastoma risk group (INRG) staging [5] and classification [6] was extracted directly from individual patient charts. To accomplish this, histologic category, grade of tumor differentiation, MYCN, 11q aberration, and ploidy information were obtained. Classification of L1 versus L2 disease was accomplished by review of the staging radiology reports: one or more reported image-defined risk factor indicated L2 disease. Clinical notes were also reviewed for staging. Where there was disagreement between radiology and the clinical note, the patient was classified as per the more advanced stage. Treatment was categorized as surgery, radiation, chemotherapy, or high-dose chemotherapy with hematopoietic stem cell rescue (HDCSR). Therapy was further classified by its intent and the line of treatment.

### 2.2. Statistical Analysis

The primary point of interest was the patients' age category, pediatric or adult, grouped by INRG classification. The primary outcome of interest was overall survival from the date of diagnosis. Secondary outcomes of interest included the effect of (neo) adjuvant radiation and chemotherapy on progression-free survival (PFS) and overall survival (OS) for stage L1 and L2 adult patients. We also examined the effect of chemotherapy choice on PFS and OS for adult patients with stage M disease. Categorical variables were compared using the Chi-squared test or the two-sided Fisher exact test. Actuarial survival was estimated using the Kaplain-Meier method and compared using the log-rank test. All statistical tests were two-sided with a *P* < 0.05 considered to be statistically significant.

## 3. Results

### 3.1. Patients

Querying the University of Texas M.D. Anderson Cancer Center's institutional database yielded 230 patients with a diagnosis of neuroblastoma or ganglioneuroblastoma. Of these, 112 patients were diagnosed before the age of 18 and were considered pediatric patients, leaving 118 adults (Table 1). The median follow-up of living pediatric patients was 6 months and 33 months for adult patients. The longest surviving pediatric patient remains living after 18 years since the date of diagnosis and the longest surviving adult has surpassed 17 years. Of the adult patients, 101 (85%) patients had localized disease, compared with 36 (33%) of pediatric patients. 78 (77%) adult patients with localized disease presented with a head and neck primary. Complete risk stratification of L1 and L2 stage adult patients was not possible: no adult patients had undergone molecular or genetic analysis of their tumors. Stage-for-stage, the category of therapies employed was comparable (Table 2). Chemotherapy agents employed included topoisomerase II inhibitors (doxorubicin and etoposide), alkylating agents (cyclophosphamide, dacarbazine, ifosfamide, melphalan, and thiotepa), platinum analogs (cisplatin, carboplatin, and oxaliplatin), spindle-cell modifiers (paclitaxel and vincristine), topoisomerase I inhibitors (topotecan), vascular endothelial growth factor small-molecule inhibitors (vandetanib and sunitinib), and immunomodulatory agents (lenalidomide and retinoid therapy) in varying combinations and dosing schedules across all stages of disease. Common regimens are listed in Table 3.

### 3.2. Outcomes

**Figure 1:**
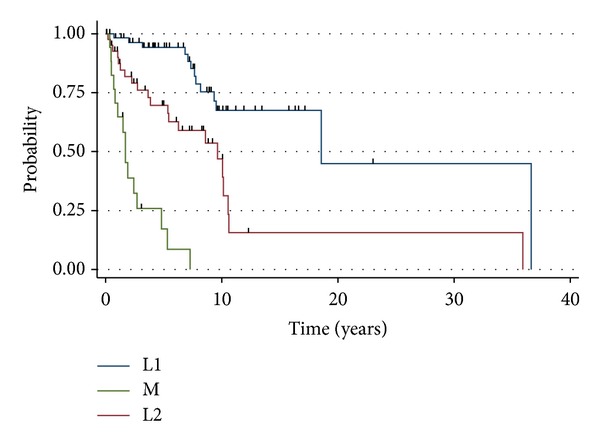
Overall survival of adult neuroblastoma patients by stage of disease. *P* < 0.001.

**Figure 2:**
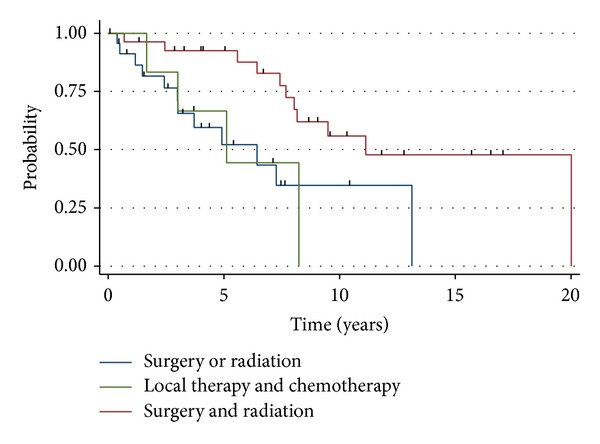
Progression-free survival for adults with L1 stage neuroblastoma. *P* = 0.07.

**Figure 3:**
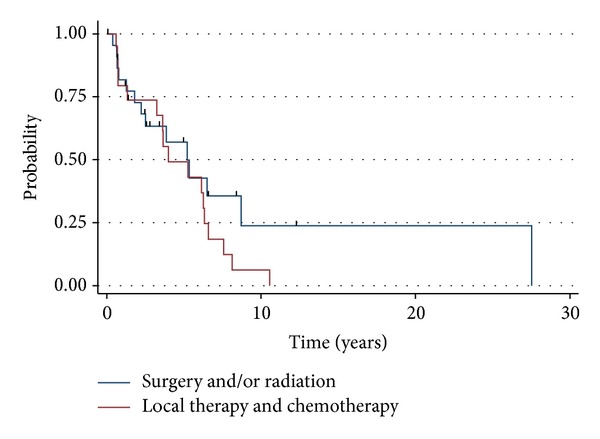
Progression-free survival for adults with L2 stage neuroblastoma. *P* = 0.23.

The median overall survival of adult patients was 18.1 years, 9.8 years, and 1.6 years for stages L1, L2, and M, respectively (Figure 1). Adults with L1 disease experienced an actuarial OS of 94%, 90%, and 69% at years 3, 5, and 10, respectively. The cohort who presented with L2 disease had an actuarial OS of 83% at 3 years, 73% at 5 years, and 41% at 10 years. Adults with M disease experienced an actuarial OS of 68%, 33%, and 13% at years 1, 2 and 5, respectively. For all stage-matched categories, the prognosis of adult neuroblastoma patients were not statistically different compared to pediatric neuroblastoma patients (L1-*P* = 0.40, L2-*P* = 0.54, and M-*P* = 0.73). For adult patients with L1 disease, the combination of surgery and radiation demonstrated a trend (*P* = 0.07) toward improved PFS (median = 11.1 months), compared with surgery or radiation alone (median = 6.4), or local therapy with chemotherapy (median = 5.1) (Figure 2). Similarly, there was no statistical difference (*P* = 0.11) in overall survival amongst patients who received surgery or radiation (median = 9.5 years), surgery and radiotherapy (median = 18.6 years), or local therapy and chemotherapy (median = 9.3 years). For adult neuroblastoma patients with L2, local therapy with surgery and radiation resulted in a median PFS of 5.2 months. The addition of chemotherapy was not associated with an increased PFS (median = 4 months; *P* = 0.23) (Figure 3). As a result of the potential for successful salvage local therapy, the median OS for local therapy and local therapy with chemotherapy was 9.6 years and 8.5 years, respectively (*P* = 0.24).

**Figure 4:**
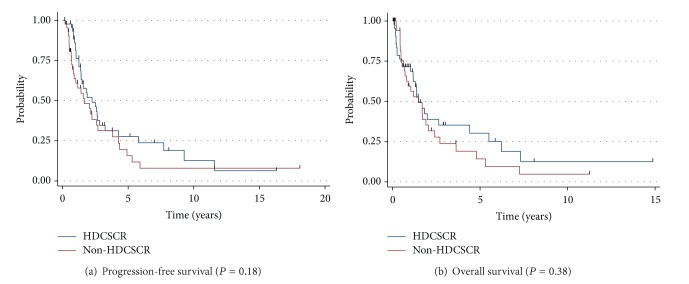
Survival of patients treated with HDCSR or non-HDCSR containing regimens.

Over the study period, 17 (14%) adult patients presented with untreated M-stage disease. Conversely, 74 (67%) of the pediatric patients presented to our center with high-risk M-stage disease. The most common chemotherapy regimens employed in this population were cisplatin and etoposide alternating with carboplatin, vincristine, and cyclophosphamide (*n* = 5, 29%); vincristine and cyclophosphamide alternating with cisplatin, doxorubicin, and dacarbazine (*n* = 4, 24%); cisplatin and etoposide only (*n* = 3, 18%); irinotecan ± temozolomide (*n* = 2, 12%). Only 1 adult M-stage patient was treated with HDCSR. Combining the entire cohort of M-stage adult (*n* = 1) and M-stage pediatric (*n* = 45) patients, patients treated with HDCSR (*n* = 46, 51%) achieved a median PFS of 1.47 years and a median OS of 2.25 years compared with a PFS of 1.48 years and an OS of 1.67 years for patients who did not undergo HDCSR (*P* = 0.18, *P* = 0.38, resp.) (Figure 4).

## 4. Discussion

Neuroblastoma may be rare in adults, but improving our empirical understanding of this disease will help guide future directions. To our knowledge, this is the largest cohorts of adult patients to be examined. Unfortunately, we were unable to properly risk-stratify early stage adult patients because of the absence of any molecular or genetic information and tissue for further analysis was not available, making comparison between patients 18 and over and under 18 years of age with L1 or L2 disease difficult. Moreover, the small number of early stage pediatric patients and similarly small number of late-stage adult patients further compromise accurate comparisons. Lastly, since we were unable to rereview all imaging with a dedicated radiologist, some early-stage patients may be misclassified despite best practice to minimize such an error.

For adult patients with L1 disease, combined surgical resection and radiotherapy seems likely to offer better PFS and OS than surgical resection alone, despite the likely scenario that patients who received combined local therapy would have more clinically concerning disease. Conversely, chemotherapy did not seem to offer any additional benefit. Similarly, almost all adult patients with L2 disease received combined surgical resection and radiotherapy, but chemotherapy was not associated with any improvement in outcomes.

Although previous studies have concluded that adult neuroblastoma has a poorer prognosis than its pediatric variant, we were unable to demonstrate a similar result. Although patients under the age of 18 years have been shown to have a better prognosis, we are unable to conclude that there is a difference in prognosis between patients younger than 18 and those 18 and older. Though, without complete staging, a conclusive result is hard to reach. We recommend that all patients of any age be risk-stratified according to the INRG risk assessment system to allow for proper determination of risk. At this time, we are unable to conclude that adult and pediatric patients have a clinically different clinical course, when stage-matched for stage, and so adult patients should be allowed entry onto protocols designed for neuroblastoma patients age > 18 months so that prospective data can be adequately and practically developed. Certainly when enrolling such patients, it would be reasonable to ensure that age is properly balanced between trial arms in the same way that patients older than 65 or 70 are managed in adult clinical trials. Subgroup analysis of such trials would allow for prospective data to be developed on whether there are real differences between adult tumor biology and the host system and whether traditionally developed pediatric regimens are reasonable for use in adult patients.

The only difference that we can observe from our data set between adult and pediatric patients is the level of enthusiasm for HDCSR. In our whole cohort, we were unable to demonstrate any benefit from such aggressive therapy. High level evidence exists for its use in the pediatric population, but, without the achievement of complete remission of metastatic neuroblastoma, we would be less eager to recommend such therapy for adults.

In summary, adult and pediatric neuroblastoma may be clinically similar diseases and we should begin accumulating evidence as if this were so.

## Figures and Tables

**Table 1:** Patient characteristics by stage of disease.

	Adult (%)	Pediatric (%)
Stage: L1	**57 (48)**	**24 (22%)**
Median age (range)	48 (18–77)	5.5 (3–15)
Histology		
Ganglioneuroblastoma	6 (11)	6 (25)
Neuroblastoma	51 (89)	18 (75)
Primary location		
Bones, skeletal system	1 (2)	1 (4)
Central nervous system	1 (2)	1 (4)
Chest cavity viscera	2 (4)	4 (17)
Head and neck	46 (81)	3 (12)
Soft tissue	2 (4)	8 (33)
Urinary tract	5 (9)	7 (29)
Stage: L2	**44 (37)**	**12 (11)**
Median age (range)	51.5 (20–81)	5.5 (3–10)
Histology		
Ganglioneuroblastoma	2 (5)	3 (25)
Neuroblastoma	42 (95)	9 (75)
Primary location		
Central nervous system	3 (7)	2 (17)
Chest cavity viscera	2 (5)	1 (8)
Head and neck	32 (73)	1 (8)
Soft tissue	3 (7)	5 (42)
Urinary tract	4 (9)	3 (25)
Stage: M	**17 (14)**	**74 (67)**
Median age (range)	29 (19–75)	5 (3–16)
Histology		
Ganglioneuroblastoma	1 (16)	4 (5)
Neuroblastoma	16 (94)	70 (95)
Primary location		
Abdominal cavity viscera	2 (12)	1 (1)
Central nervous system	0	1 (1)
Chest cavity viscera	1 (6)	5 (7)
Male genital system	1 (6)	0
Head and neck	4 (24)	2 (3)
Metastatic cancer	1 (6)	1 (1)
Soft tissue	4 (24)	25 (34)
Genitourinary tract∗	4 (24)	39 (53)

*Includes adrenal primary.

**Table 2:** Treatment by stage of disease.

	Adult (%)	Pediatric (%)	*P* value
Stage: L1	**57 (48)**	**24 (22)**	
Surgery	56 (99)	22 (92)	0.19
Radiation	31 (54)	9 (38)	0.17
Chemotherapy	3 (5)	8 (33)	0.003
HDCSR	2 (4)	5 (21)	0.02
Stage: L2	**44 (37)**	**12 (11)**	
Surgery	37 (81)	10 (83)	0.95
Radiation	32 (73)	5 (42)	0.05
Chemotherapy	21 (48)	8 (67)	0.25
HDCSR	1 (2)	1 (8)	0.35
Stage: M	**17 (14)**	**74 (67)**	
Surgery	12 (71)	58 (76)	0.62
Radiation	6 (35)	54 (71)	0.008
Chemotherapy	15 (88)	73 (96)	0.22
HDCSR	1 (7)	45 (61)	0.001

**Table 3:** First chemotherapy regimens employed in adult neuroblastoma.

First line chemotherapy regimen	*N*
Cisplatin and etoposide alternating cyclophosphamide/vincristine/carboplatin	15
Cisplatin and etoposide (+paclitaxel)	10 (+2)
Vincristine and cyclophosphamide + cisplatin/dacarbazine/adriamycin	6
Ifosfamide, carboplatin, and etoposide	3
Irinotecan and/or temozolomide	3
Ifosfamide, adriamycin, and vincristine	2
